# Association between livestock exposures and human tuberculosis in Wardha, Central India: An exploratory case-control study

**DOI:** 10.1371/journal.pone.0339024

**Published:** 2026-01-02

**Authors:** Hindol Maity, Pratibha Narang, Shilpa Moon, Rahul Narang, Abhishek Raut, Maroudam Veerasami, Sreenidhi Srinivasan, Premanshu Dandapat, Vivek Kapur, Mohan Papanna

**Affiliations:** 1 Mahatma Gandhi Institute of Medical Sciences, Wardha, Maharashtra, India; 2 Datta Meghe Institute of Higher Education and Research, Wardha, Maharashtra, India; 3 All India Institute of Medical Sciences, Bibinagar, Hyderabad, India; 4 CisGen Biotech Discoveries Pvt Ltd, Chennai, Tamil Nadu, India; 5 Huck Institute of Life Sciences, Pennsylvania State University, University Park, Pennsylvania, United States of America; 6 Indian Veterinary Research Institute, Bareilly, Uttar Pradesh, India; The University of Georgia, UNITED STATES OF AMERICA

## Abstract

**Background:**

India faces the highest burden of human and bovine tuberculosis (TB) globally. Despite this, the association between human TB and livestock exposure remains poorly understood. This exploratory study aimed to evaluate the association between human TB and livestock contact in Wardha district, Maharashtra, India.

**Methods:**

A case-control study was conducted from 01/03/2021 to 31/03/2022. Cases were microbiologically confirmed TB patients in HDSS villages, while controls were asymptomatic individuals from the same villages without TB history. Livestock in these households (HHs) were screened for TB using Interferon-Gamma-Release-Assay (IGRA), Single Cervical-Test (SCT), and Comparative-Cervical-test (CCT). Additionally, community-pooled milk samples were cultured for Mycobacterium tuberculosis complex. Fisher’s exact test was used to calculate crude odds ratios and logistic regression for adjusted odds ratios (AOR) with 95% confidence intervals (CI). A post hoc exploratory analysis to understand the relationship between effect size and sample size requirements was done.

**Results:**

The study included 52 cases and 205 controls, with a median age of 36.5 years (56% men) and 38.5 years (74% men), respectively. Analysis revealed that ownership of livestock and direct contact with cattle did not significantly alter TB risk in humans. Contact with goats showed a marginal association with human TB (AOR: 3.0; 95% CI: 1.0–9.2; p = 0.05). Of 290 livestock screened for TB, none tested positive by confirmatory tests (CCT/IGRA). While 10.2% of cattle showed reactivity to the SIT, this likely represents cross-reactivity with environmental mycobacteria. All bulk milk samples (*n* = 201) tested negative for MTBC. Post-hoc power analysis revealed that the study had limited statistical power (41%) to detect the observed association with goat contact.

**Conclusion:**

This study found no evidence of bovine TB in livestock or milk samples. While contact with goats showed a marginal association requiring further investigation, livestock ownership and raw milk consumption did not show strong associations with human TB. The absence of confirmed TB in animals suggests zoonotic transmission is not a significant contributor to human TB in this setting. The results highlight the need for larger, regionally diverse studies to better understand livestock-associated TB risk factors in India.

## Introduction

The Mycobacterium tuberculosis complex (MTBC), a group of closely related mycobacteria, is the etiological agent of tuberculosis (TB) [1], a disease that presents a significant public health challenge globally. MTBC are known for their ability to infect a wide range of hosts, leading to tuberculosis in both humans and animals, with spillovers from animals to humans resulting in zoonotic tuberculosis (zTB) [2,3]. In 2019, World Health Organization (WHO) estimated that there were 140,000 new cases of zoonotic TB worldwide, with a possible range between 69,800 and 235,000 [4], primarily caused by Mycobacterium bovis, and noted that this figure may underestimate the total burden, as other species can also cause zoonotic TB [5,6].

Despite considerable efforts to eliminate TB by 2030 [7], the role and contribution of zTB to the global human TB burden are often overlooked in most disease-endemic country settings. Hence, there are substantial knowledge gaps relating to zTB, particularly in the context of disease prevalence, transmission dynamics, and the impact on human and animal health, especially in these settings [8]. Together, these gaps undermine the development of effective intervention strategies and pose a risk to public health and to animal productivity and welfare.

With this background, WHO, the World Organisation for Animal Health, the Food and Agricultural Organization of the United Nations, and the International Union Against Tuberculosis and Lung Disease developed a ‘Roadmap’ for addressing zTB in 2017. The roadmap emphasized the need for an integrated and collaborative approach to tackle zTB and to close the knowledge gaps in disease prevalence, transmission dynamics, and impact [9].

With 2.82 million new TB cases reported in 2022, India accounted for a striking 26.6% of global TB cases making it a central player in the global fight against TB [10]. India also has the world’s largest bovine population (200 million cattle, 100 million buffaloes) [11], and a systematic review and meta-analysis from India estimates that approximately 21.8 million of these are likely infected with TB [12]. However, the relationship between livestock exposure and human TB in India remain poorly understood.

Several cross-sectional studies performed across diverse risk groups across different regions of India and utilizing a variety of diagnostic methods, present a complex picture [13–15]. Studies performed on extrapulmonary TB patients in New Delhi (2003−2007), *M. bovis* was detected in tissue samples, cerebrospinal fluid (CSF), and other body fluids, implicating zoonotic transmission [15]. In Gujarat, while a high prevalence of tuberculosis in humans and cattle was noted, no animal lineages were found in human samples [16]. However, in the neighbouring state of Maharashtra, Bapat et al. (2017), using the PCR method, reported *M. bovis* in the blood of individuals in high TB endemic areas, as well as in the blood of agricultural workers and zookeepers [17]. Further, besides *M bovis* other mycobacteria like *M orygis*, have also been reported from human cases in India [18,19]. Interestingly, recent evidence suggests more complex associations. Willgert et al. (2023) analyzed data from over 600,000 Indian households and found paradoxical protective associations between cattle ownership and human TB (OR=0.80, 95% CI: 0.71–0.89), while buffalo density showed increased risk [20]. Collectively, these studies highlight the complex and sometimes contradictory relationships between livestock and human TB in India. However, evidence remains fragmented by geography and methodology. Exploring livestock-associated exposure risks can provide insight into risk factors, even without molecular confirmation of causative organisms.

To address these knowledge gaps, we conducted a case-control study in Wardha district, Maharashtra, investigating whether livestock exposure and raw milk consumption are associated with human tuberculosis. Given conflicting evidence about livestock-TB associations in India, we sought to characterize these relationships in a high TB-burden rural setting where livestock ownership is common.

## Materials and methods

The case-control study was conducted at the Health and Demographic Surveillance Site (HDSS) of the Department of Community Medicine at the Mahatma Gandhi Institute of Medical Sciences (MGIMS), Sevagram, Wardha, India. The HDSS encompasses 96 villages with a total population of 110,000. Of these, 45% are farmers or agricultural labourers, and 31% own livestock. The livestock population in the study area consisted of cattle (n = 46,016), buffalo (n = 5,533), and goats (n = 26,466) [21]. TB care is provided to this population through the designated microscopy centres located at the Primary Health Centres, a CBNAAT (Cartridge-Based Nucleic Acid Amplification Test) centre at the district hospital functioning under National Tuberculosis Elimination Program (NTEP), and MGIMS, Sevagram. We obtained permission from the District TB Office to identify TB cases from the NTEP-NIKSHAY database on 01/03/2021.

### Study period

01/03/2021 to 31/03/2022 (local approvals obtained on 01/03/2021; development of data collection formats and consent forms between 01/03/2021–22/03/2021; and data collection between 23/03/2021 to 31/03/2022).

**Fig 1:**
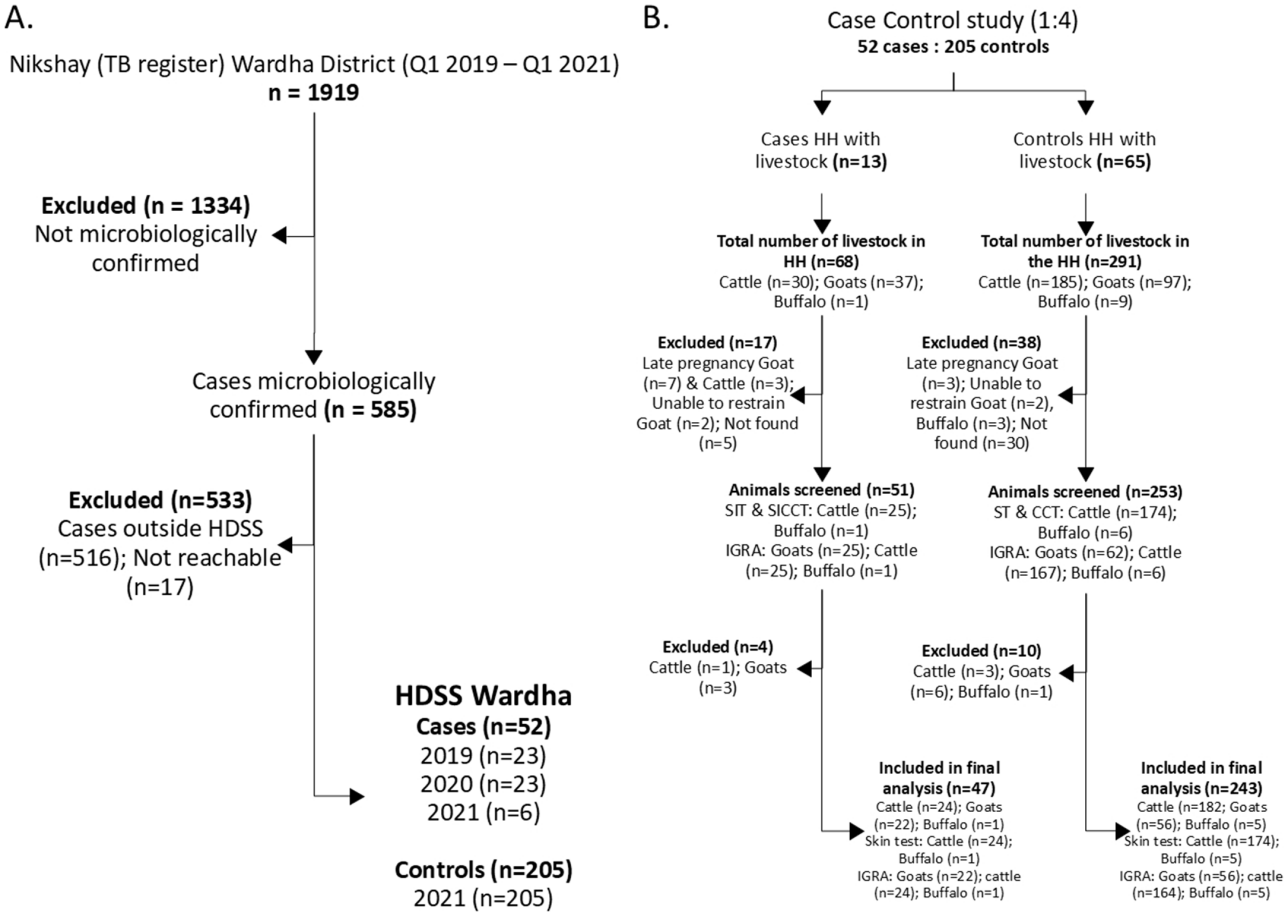
Study flow diagrams. **(A)** Selection of TB cases and controls from HDSS villages. **(B)** Livestock screening and exclusion criteria.

The study data collection was conducted in two distinct phases. **Phase I (retrospective):** Cases diagnosed between January 2019 and March 2021 were identified retrospectively from the NTEP-NIKSHAY database on 23/03/2021, while control selection and data collection occurred from March 2021 to March 2022, resulting in a 1–2-year temporal gap. A case was defined as an individual with microbiologically confirmed TB (via GeneXpert and/or bacteriological tests by NTEP labs in Wardha district) residing in the HDSS villages during the specified period. MTBC speciation was not performed on human cases as patient samples were not available due to the retrospective nature of the study. **Phase II:** From Community controls were selected from the same HDSS village as cases (Fig 1A), and livestock in case and control households were screened for TB (following Institutional animal ethics committee approval 07/07/2021) (Fig 1B). Controls were defined as persons from the same HDSS village as the cases, with no symptoms suggestive of TB (i.e., cough > 2 weeks, hemoptysis, weight loss, fever or night sweats or suspected TB by chest X-ray) or past history of TB. For selecting the controls, a list of individuals in the HDSS database belonging to the same village as cases was prepared, and random numbers were generated using RAND () function in MS Excel. For each case, four community controls were selected and were age group matched (±5 years). The study data collection and animal screening were completed on 31/03/2022. Livestock exposure was defined as any physical interaction involving handling, feeding, milking, cleaning, assisting with births, administering medication or vaccinations, or other forms of close proximity where the individual touches or is exposed to secretions, excretions, or respiratory emissions of the livestock.

### Sample size

To establish an appropriate sample size for the exploratory studies, we estimated statistical power using the Fleiss method implemented in EpiInfo [22]. This enabled us to consider the impact of various expected effect sizes and the anticipated prevalence of exposure among the control group. Not having any prior knowledge of prevalence in either group, we opted for a sample size comprising 51 cases and 203 controls, maintaining a 1:4 ratio to achieve an 80% power for detecting an effect size, represented as an odds ratio of 2.5, under the assumption that 50% of the control group had exposure to livestock based on a confidence interval of 95%. The sample size and power calculations for the exploratory analyses aimed to strike a balance between feasibility and the ability to detect meaningful associations between TB and exposure to livestock in the target population.

For post-hoc analyses, to evaluate the impact of effect size on the power of the study, we performed a series of sample size calculations. These calculations were based on the desired power level of 80% and a significance level of 5%, across a range of baseline proportions from 5% to 50%. The effect sizes considered were absolute differences in proportions of 5%, 10%, 15%, and 20% between case and control groups. The required sample sizes were computed using the ‘pwr’ package in R, which employs the Cohen’s h statistic to translate the effect size into a standardized measure and then estimates the required sample size for a two-proportion z-test [23]. Calculations assumed equal sample sizes in both case and control groups and a two-sided hypothesis test.

### Screening of livestock for TB reactivity

TB reactivity screening in livestock was performed using both the SCT and CCT. The procedure entailed preparing two distinct injection sites on each animal’s neck, spaced 12 cm apart. McLintock syringes were used to administer 0.1 ml of avian purified protein derivative A (strain D4ER; PPD-A; 2500 IU) on the upper site and bovine PPD-B (strain AN5; PPD-B; 3000 IU) (Prionics, Switzerland) at the lower site, as per established protocols. Skin thickness before administration of PPDs was measured in millimetres (0-h value) using a Vernier calliper. The same operator measured skin thickness again at 72 hours. The increase in thickness at the bovine PPD-B site gave the reading for SIT, and the response to the avian PPD injection served as a control to correct for non-specific reactivity. The CCT test was considered positive if the reaction to PPD-B minus PPD-A exceeded 4 mm, indicating a presumptive diagnosis of TB infection in the livestock. This method is in accordance with the guidelines set forth by the OIE (World Organisation for Animal Health) [24].

Additionally, we performed using commercially available IGRA kits (BOVIGAMTM Prionics), which is an in vitro blood-based assay that measures gamma-interferon (IFN-γ) production in response to bovine PPD. Blood samples collected into heparinized tubes from the jugular vein of each animal and were transported to the MGIMS laboratory within 4 hours at temperatures maintained between 18–21°C and were processed within 8 hours of collection. The whole blood samples were incubated with bovine PPD, avian PPD, and a mitogen control (phytohaemagglutinin) in separate wells of a 32-well culture plate. After 16–24 hours of incubation, the plasma was harvested, and the concentration of IFN-γ was determined using BOVIGAM ELISA. The sample was considered positive if the optical density (OD at 450 nm) values in response to bovine PPD were derived to be higher than those for avian PPD and the negative control [25,26].

### Bulk milk sample testing

Pooled milk samples were gathered from two community collection centres receiving cattle milk from 46 villages that included the milk from cases and controls households between March-April 2021. These community-level samples could not be linked to specific households, limiting household-specific risk assessment. We utilized Eppendorf sterile tubes to collect 15 ml of milk from each of the above containers (n = 78) and ensured their transportation to the MGIMS laboratory within 2–3 hours, maintaining a temperature range of 4–8°C [27]. Upon arrival, milk samples were processed using a standardized Hexadecyl pyridinium Chloride (HPC) decontamination protocol optimized for isolating *Mycobacterium bovis* and other members of the *Mycobacterium tuberculosis* complex (MTBC). Briefly, 15 mL of milk was collected in sterile containers, and the exterior of each bottle was disinfected with 70% ethanol to prevent contamination. Samples were centrifuged at 4,000 × g for 15 minutes, and the resulting cell layer was washed twice with sterile phosphate-buffered saline (PBS) to remove residual milk fat. The pellet was resuspended in 10 mL of 0.75% (w/v) HPC (Sigma) and incubated at room temperature (21°C) for 30 minutes. After decontamination, the samples were centrifuged again (4,000 × g for 15 minutes), and the pellet was resuspended in 2 mL of sterile PBS. From this suspension, 500 µL was inoculated onto each of three Lowenstein–Jensen (LJ) slants—two containing 0.5% sodium pyruvate and one containing glycerol and two MGIT (with OADC – PANTA). Inoculated LJ slants and MGIT were incubated at 37°C for up to six weeks, with weekly monitoring for microbial growth or contamination.

### QC spiking

The technique of decontamination was standardized for quality assurance by using a vaccine strain of *M. bovis* BCG as a positive control. This involved spiking the milk sample, which underwent the same HPC treatment method [28] for 30 minutes as the test samples. Commercial pasteurised milk was used as the negative control. Pasteurised milk aliquots were spiked with ~100 CFU of *M. bovis* BCG vaccine strain per 15 ml and processed in parallel: (A) untreated control, (B) 0.75% HPC for 5 hours at room temperature, and (C) 0.75% HPC for 30 minutes. Results were enumerated on Löwenstein–Jensen media.

### Data collection

Amidst the COVID-19 pandemic, we adapted our methodology to conduct telephonic interviews with cases and controls using a semi-structured questionnaire. Verbal consents were obtained to collect data encompassing demographics (age, sex, and occupation), livestock ownership, details about the livestock, contact with livestock, and consumption of raw milk). This information was later verified, and informed consent was obtained during home visits for animal TB screening. Livestock exposure was defined as any physical interaction involving handling, feeding, milking, cleaning, assisting with births, administering medication or vaccinations, or other forms of close proximity with potential exposure to secretions, excretions, or respiratory emissions. No biological samples were collected from human participants.

For data entry and analysis, we utilized Epi Info 7.2, GraphPad Prism software, and using R version 4.2.3, with the analysis code provided as supplementary material (S1 and S2 Files). The analysis included descriptive statistics, presented as frequencies, medians with ranges, and proportions. Due to rare events (particularly raw milk consumption with only 4 exposed individuals), Fisher’s exact test was used to calculate crude odds ratios with exact 95% confidence intervals for all exposures. Variables with p < 0.2 in univariate analysis were included in logistic regression models, performed only for exposures with ≥3 exposed cases to ensure model stability. Model diagnostics included Hosmer-Lemeshow goodness-of-fit test, variance inflation factors for multicollinearity, and C-statistic for discrimination. Post-hoc power analyses were conducted using Cohen’s h statistic.

### Ethical clearances

Ethical approvals were obtained from the Institutional Ethics Committee and Institutional Animal Ethics Committee (IRB: MICR/187/2021 & 262B/2021) at MGIMS Wardha, India and the national Committee for the Purpose of Control and Supervision of Experiments on Animals’ (Ref: 700/2021WARDHA). Permissions were obtained from Wardha District Tuberculosis Unit and District Animal Husbandry Department. Participation in the study was voluntary and written informed consent was obtained from study participants before the interviews.

## Results

### Study population

During the study period, 1,919 TB cases were reported in Wardha district, of which 585 (30%) were microbiologically confirmed. Of these, 52 cases residing in HDSS villages met inclusion criteria and were enrolled, along with 205 age-matched controls from the same villages (Fig 1A). The characteristics of the study population are presented in Table 1. The median age was 36.5 years (range: 9–80) for cases and 38.5 years (range: 14–80) for controls. Males comprised 55.8% (29/52) of cases and 74.5% (152/204) of controls.

**Table 1:** Characteristics of tuberculosis cases and controls in Wardha district, India (2019-2022)*.

Characteristic	Cases (*n* = 52)	Controls (*n* = 204)
**Demographics**		
Age, Median (Range)	36.5 (9-80)	38.5 (14-80)
Male Sex, *n* (%)	29 (55.8)	152 (74.5)
**Livestock Ownership, *n* (%)**		
Any Livestock	13 (25.0)	65 (31.9)
Cattle	10 (19.2)	55 (27.0)
Goats	7 (13.5)	12 (5.9)
**Animal Contact, *n*, (%)**		
Any Livestock Contact	14 (26.9)	69 (33.8)
Cattle Contact	7 (13.5)	53 (26.0)
Goat Contact	6 (11.5)	9 (4.4)
**Dietary Exposure, *n* (%)**		
Raw Milk Consumption	2 (3.8)	2 (1.0)

**Note**. Data presented as *n* (%) unless otherwise specified. Cases were microbiologically confirmed TB patients identified from NTEP-NIKSHAY database (2019-2021). Controls were randomly selected from the same villages and age-matched (±5 years). Missing data: one control excluded from sex analysis.

Livestock ownership was reported by 25% (13/52) of cases and 31.9% (65/204) of controls. Among specific animals, cattle ownership was reported by 19.2% of cases and 27% of controls, while goat ownership was more common among cases (13.5%) than controls (5.9%). Direct contact with any livestock was similar between groups (26.9% of cases vs 33.8% of controls), though contact with goats specifically was reported by 11.5% of cases compared to 4.4% of controls. Raw milk consumption was rare in both groups, reported by only 3.8% (2/52) of cases and 1.0% (2/204) of controls.

### Absence of TB in livestock and milk samples

**Fig 2:**
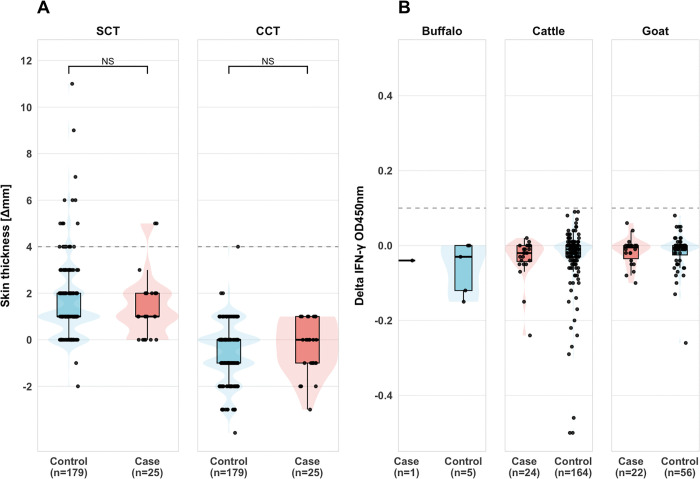
Tuberculosis test results in livestock. **(A)** Single Cervical Test (SCT) and Comparative Cervical Test (CCT) results in cattle from case and control households. **(B)** Interferon-gamma release assay (IGRA) results for all livestock species.

To assess potential zoonotic transmission risk, we screened 290 livestock from case and control households for TB (Fig 1B). While 21 of 206 cattle (10.2%, 95% CI: 6.8–15.1%) showed reactivity to the single intradermal test (SIT), none were confirmed positive by the more specific comparative test (CCT) or IGRA, suggesting cross-reactivity with environmental mycobacteria rather than true TB infection (Fig 2). The distribution of SIT-reactive cattle did not differ significantly between case households (2/24, 8.3%) and control households (19/182, 10.4%; p = 1.0). Additionally, all 201 pooled milk samples collected from community centers serving both case and control villages tested negative for MTBC despite quality control confirmation using BCG-spiked samples. The complete absence of confirmed TB in livestock and milk indicates that direct animal-to-human transmission was not occurring in this setting.

In the QC experiment with ~100 CFU input in pasteurised milk, the untreated control produced 72 colonies (72% recovery), the 0.75% HPC × 5 h treatment produced 28 colonies (28% recovery; 38.9% of the untreated control), and the 0.75% HPC × 30 min treatment produced 43 colonies (43% recovery; 59.7% of the untreated control).

### Livestock exposure patterns and human TB

Given the absence of TB in animals, we examined whether livestock exposure patterns differed between TB cases and controls (Table 2). Using Fisher’s exact test for all crude associations, we found no statistically significant differences at the p < 0.05 threshold. Contact with goats showed the strongest association with TB (OR=2.81, 95% CI: 0.78–9.37, p = 0.089), which reached marginal significance after adjustment for age and sex (aOR=3.03, 95% CI: 1.00–9.17, p = 0.050). Goat ownership showed a similar pattern (crude OR=2.48, 95% CI: 0.78–7.29, p = 0.076; AOR = 2.66, 95% CI: 0.81–8.73, p = 0.107).

**Table 2:** Crude and adjusted odds ratios for associations between livestock exposures and human tuberculosis in Wardha district, India (2019-2022).

Exposure	Cases/Total (%)	Controls/Total (%)	Crude OR (95% CI)	P-Value	Adjusted OR^a^ (95% CI)	P-Value
**Animal Contact**						
Goat Contact	6/52 (11.5)	9/204 (4.4)	2.81 (0.78-9.37)	0.09	3.03 (1.00-9.17)	0.05
Cattle Contact	7/52 (13.5)	53/204 (26.0)	0.44 (0.16-1.07)	0.07	0.48 (0.13–1.77)^b^	0.27
**Animal Ownership**						
Goat Ownership	7/52 (13.5)	12/204 (5.9)	2.48 (0.78-7.29)	0.08	2.66 (0.81-8.73)	0.11
Cattle Ownership	10/52 (19.2)	55/204 (27.0)	0.65 (0.27-1.42)	0.29	0.69 (0.20–2.41)^b^	0.56
Any Livestock Ownership	13/52 (25.0)	65/204 (31.9)	0.71 (0.33-1.48)	0.40	0.72 (0.34-1.53)	0.39
**Dietary Exposure**						
Raw Milk Consumption	2/52 (3.8)	2/204 (1.0)	4.01 (0.28-56.5)	0.18	-^c^	–

**Footnotes:** ^a^Adjusted for age and sex; ^b^From full multivariable model including all livestock exposures; ^c^Not calculated due to insufficient exposed cases (n = 2). CI: Confidence interval; OR: Odds ratio. Crude odds ratios calculated using Fisher’s exact test due to small cell counts. P-values <0.05 were considered statistically significant. One control had missing data for demographic variables, resulting in *n* = 204 for exposure analyses.

Several livestock exposures showed protective trends that did not reach statistical significance: cattle contact (OR=0.44, 95% CI: 0.16–1.07, p = 0.07), cattle ownership (OR=0.65, 95% CI: 0.27–1.42, p = 0.29), and any livestock ownership (OR=0.71, 95% CI: 0.33–1.48, p = 0.40). These protective trends likely reflect unmeasured confounding or chance findings given our limited statistical power. Raw milk consumption could not be reliably assessed due to extreme rarity of exposure, with only 2 cases (3.8%) and 2 controls (1.0%) reporting consumption (OR=4.01, 95% CI: 0.28–56.52, p = 0.18).

The multivariable model including all livestock exposures demonstrated acceptable fit (Hosmer-Lemeshow p = 0.84) with no evidence of multicollinearity (all VIF values <3.4) but limited discriminative ability (C-statistic = 0.66) (S1 Table). Full model coefficients are provided in (S2 Table). Post-hoc power analysis revealed our study achieved 39–41% statistical power for goat-related exposures, 31% for cattle contact, and only 17% for overall livestock ownership (S3 Table). Raw milk consumption had less than 10% power with only 4 exposed individual’s total. These power limitations, combined with wide confidence intervals for most associations, indicate our findings should be interpreted as exploratory rather than definitive.

## Discussion

This case-control study found no evidence of MTBC in livestock or milk samples from households with and without human TB cases in rural Wardha district. Of 290 livestock screened using multiple diagnostic methods, none tested positive by confirmatory tests (CCT or IGRA), and all 201 pooled milk samples were culture-negative for MTBC. While 10.2% of cattle showed reactivity to the single intradermal test, the absence of confirmation by comparative testing suggests cross-reactivity with environmental mycobacteria rather than true MTBC infection [29]. These findings indicate that direct animal-to-human transmission of TB was not occurring in our study population during the sampling period. Consequently, while we examined associations between livestock exposure and human TB, we cannot assess zoonotic transmission risk in the absence of infected animals, only whether livestock contact patterns differ between TB patients and healthy controls for reasons unrelated to animal infection.

Despite the absence of TB in livestock, we observed associations between certain animal exposures and human TB that merit examination. The marginal association between goat contact and human TB (AOR = 3.03, 95% CI: 1.00–9.17, p = 0.05) requires cautious interpretation. This finding, based on only 6 exposed cases and 9 exposed controls, achieved just 32% statistical power and has a confidence interval barely excluding 1.0. While we found no TB infection in the 78 goats tested, the 1–2-year gap between case identification and testing means we likely examined different animals than those present during disease development. Nevertheless, several factors suggest this association may not be causal. First, with multiple exposures tested and no correction for multiple comparisons, this p = 0.05 finding could represent Type I error [30]. Second, unmeasured confounding may explain the association; goat ownership in this region may correlate with specific occupational, socioeconomic, or environmental factors not captured by our binary exposure variables. Third, the absence of any TB-positive goats during our sampling suggests low or absent infection pressure. Given these limitations, this finding should be considered hypothesis-generating, warranting longitudinal studies with contemporaneous animal testing.

Conversely, several livestock exposures showed non-significant protective trends, with cattle contact demonstrating the strongest inverse association (OR=0.44, 95% CI: 0.16–1.07, p = 0.067). These findings align remarkably with recent evidence from Willgert et al. (2023), who analyzed over 600,000 households across India and reported similar protective associations for cattle density (OR=0.80, 95% CI: 0.71–0.89) and bovine ownership (OR=0.94, 95% CI: 0.90–0.99). The consistency of these paradoxical protective effects across different scales and methodologies suggests they reflect real epidemiological patterns rather than study artifacts. Several mechanisms could explain these inverse associations: livestock ownership may serve as a proxy for better socioeconomic status or nutritional security [31], regular animal contact might indicate better overall health (a “healthy worker effect”) [32], or reverse causation may occur if individuals developing TB symptoms reduce animal contact or sell livestock to cover medical expenses [33]. The absence of TB in our livestock population strengthens the interpretation that these associations reflect socioeconomic or behavioral factors rather than any protective immunological mechanism.

Our findings contribute to the complex landscape of livestock-TB associations reported across India. While studies from Delhi [14] and Maharashtra [17] have detected *M. bovis* in human clinical samples, we found no evidence of animal infection despite testing 290 livestock. This geographic heterogeneity likely reflects regional differences in animal TB prevalence, livestock management practices, and dairy processing methods [12]. The absence of bovine TB in our study area contrasts with the 7.3% national prevalence reported by Srinivasan et al. (2018), though Maharashtra’s specific prevalence of 2.7% is closer to our findings. Wardha district may represent a low-risk area for zoonotic transmission due to smaller herd sizes, less intensive farming, or limited animal movement compared to peri-urban dairy operations. This regional variation underscores the importance of local TB surveillance in both human and animal populations [9].

The HPC decontamination method used in our study for isolating *M. bovis* or *M. tuberculosis* may have contributed to the negative culture results. However, by employing a shortened 30-minute exposure, we achieved improved recovery (43 colonies; 40% reduction compared to untreated samples) relative to the standard 5-hour exposure, which markedly decreases viable counts (72 → 28 colonies; 61% loss). Therefore, we are confident that our approach provided effective decontamination while maintaining acceptable recovery of mycobacteria.

Our study has important limitations. We could not perform MTBC speciation on human cases due to the retrospective design, preventing definitive exclusion of zoonotic transmission. The 1–2-year gap between case identification and data collection introduces recall bias and temporal misalignment of exposures. The study was severely underpowered (27–32% power for main exposures), limiting confidence in both positive and negative findings. Pooled milk samples could not be linked to specific households, and binary exposure measures failed to capture contact intensity or duration. Despite these limitations, several strengths support our conclusions: multiple diagnostic tests for livestock TB, population-based sampling through the HDSS platform, simultaneous examination of humans and animals, 95% livestock screening coverage, and validated milk culture methods with quality controls.

Our findings indicate that zoonotic TB is not a public health priority in Wardha district, with human-to-human transmission likely driving the TB burden given the complete absence of TB in livestock and milk. While goat contact showed a marginal association with human TB, the lack of infection in goats, low statistical power, and borderline significance suggest this finding reflects unmeasured confounding or chance rather than true risk. The protective trends for cattle ownership align with emerging Indian evidence of complex, non-causal relationships between livestock and TB, likely reflecting socioeconomic factors. Future studies should employ prospective designs with MTBC speciation, contemporaneous exposure assessment, and adequate power to clarify these relationships. Until evidence of bovine TB emerges in this region, public health efforts should focus on human-to-human transmission rather than zoonotic pathways.

## Supporting information

S1 Methods Detailed statistical methods, including exact logistic regression approach and handling of sparse data. (DOCX)

S1 Table Model diagnostics, including Hosmer-Lemeshow test, variance inflation factors, and C-statistic. (CSV)

S2 Table Full multivariable logistic regression model coefficients. (CSV)

S3 Table Post-hoc power analysis for primary exposure variables. (CSV)

S1 File R script for case-control study analysis. (TXT)

S2 File R script for animal study analysis. (R)

S1 Dataset De-identified case-control dataset. (XLSX)

S2 Dataset Livestock screening dataset. (XLSX)
