# Supplementary material for: Association between livestock exposures and human tuberculosis in Wardha, Central India: An exploratory case-control study
Source: PLoS One. 2026 Jan 2;21(1):e0339024. doi: 10.1371/journal.pone.0339024 (PMC12758689; doi:10.1371/journal.pone.0339024)
Supplement: S1 Methods — (DOCX) [file pone.0339024.s001.docx]

## **Supplementary Methods**

### **S1. Study Setting and Temporal Considerations**

The retrospective case identification period (January 2019 - March 2021) preceded control selection and data collection (March 2021 - March 2022) by 1-2 years. This temporal gap represents a limitation acknowledged in our sensitivity analyses. The HDSS population includes 46,016 cattle, 5,533 buffalo, and 26,466 goats based on district livestock census data.

### **S2. Sample Size Determination**

Sample size was calculated using the Fleiss method with the following assumptions:

- 1:4 case-to-control ratio
- 50% exposure prevalence in controls (conservative estimate given lack of prior data)
- 80% power to detect OR ≥2.5
- Type I error rate of 5%

This yielded a target of 51 cases and 204 controls. The final sample included 52 cases and 205 controls after excluding 533 cases who were outside HDSS boundaries or unreachable.

### **S3. Exposure Assessment**

Livestock exposure was defined as any physical interaction involving handling, feeding, milking, cleaning, assisting with births, administering medication/vaccinations, or close proximity with potential exposure to secretions, excretions, or respiratory emissions. Exposures were collected as binary variables (yes/no) with additional frequency data (daily/weekly/occasionally/never) for sensitivity analyses.

### **S4. Detailed Statistical Methods**

#### 4.1 Data Cleaning and Preparation

- Missing values coded as "NA" were converted to R's NA type
- Exposure variables verified for biological plausibility
- Complete case analysis performed for primary outcomes

#### 4.2 Exact Methods for Rare Events

Given that raw milk consumption (n=4 exposed) and several other exposures had <5 events, we used Fisher's exact test for all crude associations. This provides:

- Exact p-values not dependent on large-sample approximations
- Exact confidence intervals using the conditional maximum likelihood estimate
- Valid inference even with zero cells in 2×2 tables

#### 4.3 Multivariable Adjustment

For exposures with ≥3 exposed cases, we fitted logistic regression models:

logit(P(TB)) = β₀ + β₁(Exposure) + β₂(Age) + β₃(Sex)

For exposures with <3 exposed cases, adjusted analyses were not performed due to model instability.

#### 4.4 Model Diagnostics

- **Goodness-of-fit**: Hosmer-Lemeshow test with 8 groups (reduced from default 10 due to sample size)
- **Multicollinearity**: VIF calculated for all predictors; VIF >5 indicating concern
- **Discrimination**: C-statistic (area under ROC curve) with interpretation: <0.7 poor, 0.7-0.8 acceptable, >0.8 excellent
- **Sample size adequacy**: Events per variable (EPV) ratio calculated; EPV <10 suggesting potential overfitting

#### 4.5 Power Analysis

Post-hoc power calculated using:

- Observed proportions in cases and controls
- Two-sided tests at α=0.05
- Sample size requirements for 80% power estimated using the formula:
- n = 2(Z_α + Z_β)²p(1-p)/δ²
- where δ is the observed difference in proportions and p is the pooled proportion

### **S5. Software and Reproducibility**

All analyses were performed using R version 4.2.3 with the following packages:

- tidyverse (v2.0.0): Data manipulation
- epitools (v0.5-10.1): Epidemiological calculations and exact tests
- ResourceSelection (v0.3-5): Hosmer-Lemeshow test
- car (v3.1-2): Variance inflation factors
- pROC (v1.18.0): ROC curve analysis

The complete R script is provided as Supplementary File S1, enabling full reproduction of all analyses. Data are available as Supplementary File S2 (with participant identifiers removed).

### **S6. Reporting Guidelines**

This study follows STROBE (Strengthening the Reporting of Observational Studies in Epidemiology) guidelines for case-control studies. The STROBE checklist is provided as Supplementary Table S4.

### **S7. Limitations Addressed in Analysis**

Our analytical approach explicitly addressed several limitations:

1. **Rare events**: Used exact methods rather than large-sample approximations
2. **Small sample size**: Reported power calculations and EPV ratios
3. **Temporal bias**: Acknowledged 1-2 year gap between case identification and control selection
4. **Multiple comparisons**: Did not adjust for multiple testing given exploratory nature, but reported all analyses performed

### **S8. Deviations from Protocol**

The following deviations occurred due to practical constraints:

1. Telephone interviews replaced in-person interviews (COVID-19 restrictions)
2. Pooled milk samples used instead of household-specific samples (logistical constraints)
3. MTBC speciation not performed on human cases (samples not available retrospectively)
